# Comparative analysis of the efficacy of oblique lateral interbody fusion versus transforaminal lumbar interbody fusion in the treatment of lumbar disc herniation

**DOI:** 10.1038/s41598-024-81261-w

**Published:** 2024-11-27

**Authors:** Daodong Chen, Tao Liu, Kunyang Du, Zhenjun Zhu

**Affiliations:** Department of Orthopedics, Xinxiang Central Hospital, The Fourth Clinical College of Xinxiang Medical University, 56 Jinsui Avenue, Xinxiang, 453000 Henan China

**Keywords:** Lumbar disc herniation, Oblique lateral Interbody Fusion, Transforaminal lumbar Interbody Fusion, Afusion rate, Postoperative recovery, Anatomy, Fracture repair

## Abstract

Lumbar disc herniation (LDH) often necessitates surgical intervention when conservative treatments fail. Oblique Lateral Interbody Fusion (OLIF) and Transforaminal Lumbar Interbody Fusion (TLIF) are two commonly used techniques for treating LDH, each offering distinct surgical approaches. This study aimed to compare the efficacy of OLIF versus TLIF in terms of pain relief, functional outcomes, spinal alignment correction, fusion success, and postoperative recovery. A retrospective study was conducted on 133 patients who underwent either OLIF (*n* = 68) or TLIF (*n* = 65) between January 2020 and December 2022. Data on patient demographics, pain and functional outcomes (measured by Visual Analogue Scale [VAS] and Oswestry Disability Index [ODI]), radiological outcomes (sagittal and coronal Cobb angles, apical vertebra deviation), fusion and collapse rates, and postoperative recovery (ambulation time and hospitalization duration) were collected. Statistical analysis was performed using t-tests and chi-square tests, with significance set at *P* < 0.05. Both groups demonstrated significant postoperative improvements in pain and functional outcomes. OLIF resulted in significantly better long-term pain reduction (VAS 1.99 ± 0.67 vs. 2.29 ± 0.92, *P* = 0.03) and greater spinal alignment correction, particularly in sagittal Cobb angle and apical vertebra deviation (*P* < 0.001). The fusion rate was similar between OLIF (97.92%) and TLIF (96.61%, *P* = 0.56), but OLIF had a lower collapse rate (8.33% vs. 18.64%, *P* < 0.001). OLIF also facilitated faster postoperative recovery, with earlier ambulation and shorter hospitalization time (*P* < 0.001 for both). While both OLIF and TLIF are effective for treating LDH, OLIF offers superior long-term pain relief, better spinal alignment correction, reduced collapse rates, and faster recovery. These findings suggest that OLIF may be a more advantageous option for patients requiring lumbar interbody fusion.

## Introduction

Lumbar disc herniation (LDH) is a prevalent condition affecting the lumbar spine, often leading to significant pain, disability, and decreased quality of life in affected individuals. LDH occurs when the intervertebral disc, which acts as a cushion between the vertebrae, experiences structural changes, such as the protrusion or extrusion of its nucleus pulposus, leading to nerve root compression. This condition typically results in radicular symptoms such as sciatica, lower back pain, and motor or sensory deficits^1–3^. Conservative treatment options, including physical therapy, pharmacological intervention, and epidural steroid injections, are often the first line of treatment. However, surgical intervention becomes necessary in patients who fail to respond to these therapies, particularly when neurological deficits or intractable pain persist. Among the various surgical options, lumbar interbody fusion has become a standard treatment for patients with LDH. In recent years, two major techniques have emerged as the primary approaches to lumbar interbody fusion: Oblique Lateral Interbody Fusion (OLIF) and Transforaminal Lumbar Interbody Fusion (TLIF)^4,5^. These techniques have shown promising results in achieving pain relief, improving functional outcomes, and providing spinal stability by fusing the affected vertebrae. However, there remains a significant debate regarding the superiority of one technique over the other in terms of efficacy, safety, and long-term outcomes.

The Oblique Lateral Interbody Fusion (OLIF) technique, introduced as a minimally invasive surgical option, has gained attention due to its potential advantages in reducing muscle damage, blood loss, and postoperative recovery time. By approaching the lumbar spine obliquely through a lateral incision, OLIF allows access to the disc space without the need for direct manipulation of the spinal canal, which reduces the risk of nerve injury^6,7^. Furthermore, OLIF offers the benefit of preserving the posterior musculature and spinal structures, theoretically leading to faster recovery and fewer postoperative complications. Nonetheless, OLIF is associated with certain limitations, such as the risk of injury to the abdominal vessels and the lumbar plexus during the approach^8,9^. In contrast, the Transforaminal Lumbar Interbody Fusion (TLIF) technique has been widely utilized for several decades and remains a popular choice among spine surgeons. TLIF is performed via a posterior approach, allowing direct visualization and access to the intervertebral disc space. By removing the damaged disc and replacing it with an interbody cage, TLIF provides effective decompression of neural elements and promotes fusion between vertebrae^10–12^. Despite its advantages, TLIF is a more invasive procedure compared to OLIF, potentially resulting in increased muscle dissection, greater blood loss, and prolonged recovery. However, the ability to achieve solid fusion with TLIF, coupled with the extensive clinical experience supporting its efficacy, continues to make it a viable option for treating LDH.

This comparative study aims to evaluate the efficacy of OLIF versus TLIF in the treatment of lumbar disc herniation. By examining postoperative outcomes, complication rates, and patient-reported measures of pain and function, this analysis seeks to provide a comprehensive understanding of the benefits and limitations of each technique. Ultimately, the goal is to inform clinical decision-making and optimize treatment strategies for patients suffering from LDH.

## Methods

### Study design

A comprehensive retrospective study was conducted at our hospital to evaluate the efficacy of OLIF versus TLIF in the treatment of LDH. The study encompassed patients treated between January 2020 and December 2022. Patients who underwent OLIF were classified into the OLIF group (*n* = 68), while those who received TLIF were categorized into the TLIF group (*n* = 65). The study design, research objectives, and protocols adhered to the Strengthening the Reporting of Observational Studies in Epidemiology (STROBE) guidelines^13^. Ethical approval for the study was obtained from the Ethics Committee of our hospital. Informed consent was obtained from all subjects and/or their legal guardian(s). The research design, objectives, and protocols were rigorously reviewed and approved by the ethics committee of our hospital, ensuring compliance with established guidelines and regulations. This study was conducted in strict accordance with the ethical standards of the Declaration of Helsinki for medical research involving human subjects. To safeguard participant privacy, all data were handled confidentially, and personal identifiers were removed from the dataset prior to analysis.

### Inclusion and exclusion criteria

*Inclusion vriteria*:

1. Patients aged between 18 and 75 years with a confirmed diagnosis of LDH based on clinical symptoms and radiological findings (MRI and/or CT scan).
2. Patients who underwent either OLIF or TLIF between January 2020 and December 2022.
3. Patients with single-level lumbar disc herniation requiring surgical intervention.
4. Patients with a follow-up period of at least 12 months postoperatively.

*Exclusion criteria*:

1. Patients with previous lumbar spine surgery.
2. Patients with multilevel degenerative disc disease or other spinal pathologies, such as spinal tumors, infections, or severe spinal deformities.
3. Patients with significant osteoporosis or other metabolic bone diseases that could affect the outcomes of spinal fusion surgery.
4. Patients with incomplete medical records or inadequate follow-up data.

### OLIF group procedure

For the OLIF group, general anesthesia with endotracheal intubation was administered, and patients were positioned in the right lateral decubitus position, with the torso securely stabilized. Preoperative lateral X-rays were used to identify and mark the target lumbar disc level. Following standard antiseptic preparation and continuous ECG monitoring, a 3 cm incision was made approximately 6–7 cm anterior to the midline of the marked intervertebral space. Sequential incisions were made through the skin, subcutaneous tissue, and external oblique aponeurosis, followed by blunt dissection of the external oblique, internal oblique, and transversus abdominis muscles. Blunt dissection continued through the peritoneal space to isolate the aorta and psoas major muscle. Upon reaching the anterolateral side of the target disc space, the segment was confirmed with fluoroscopic guidance. A retractor was placed, and the annulus fibrosus was exposed. A spinal endoscope was inserted, and the herniated disc material was removed under fluoroscopy. The annulus and posterior longitudinal ligament were treated with radiofrequency ablation, ensuring thorough decompression. After preparing the disc space by excising the annulus and removing disc material, a suitable interbody cage was inserted under fluoroscopic guidance, followed by pedicle screw or lateral plate fixation through the OLIF approach to stabilize the segment.

### TLIF group procedure

In the TLIF group, patients were placed in the prone position under general anesthesia with endotracheal intubation. The torso was stabilized, and lumbar imaging was used to identify the surgical level. A 3 cm incision was made parallel to the midline at the marked level, and the skin and fascia were incised sequentially. A tubular retractor was placed, and the spinal endoscope was introduced. The facet joint and ligamentum flavum were resected, exposing the nerve root and dura, which were carefully protected. The annulus fibrosus was incised, and disc material was removed with curettes and rongeurs. The intervertebral space was cleared, and an interbody cage was trialed and confirmed under fluoroscopy. Bone graft was placed into the disc space, and the definitive cage was inserted. Bilateral pedicle screws were inserted, and titanium rods were affixed to secure the construct. Proper placement was confirmed fluoroscopically before closing the incision in layers.

To minimize variability and reduce bias related to differences in surgical technique and experience, all surgeries in both groups were performed by the same surgical team, led by a single experienced surgeon. While involving multiple surgeons could theoretically enhance generalizability, it would also introduce variability in technique, potentially confounding the study’s findings. Standardizing the surgical team ensures that observed differences between OLIF and TLIF outcomes are attributable to the surgical approach rather than inter-surgeon variability, thereby enhancing the internal validity of our results. Both groups received standardized postoperative care, including antibiotic therapy and fluid replacement. Additionally, all patients were instructed to wear a lumbar brace for three months post-surgery, to support the spine and facilitate safe mobilization when getting out of bed during the recovery period.

### Data collection

Pain Assessment: Pain levels were evaluated using the Visual Analogue Scale (VAS), which scores pain intensity on a scale from 0 to 10. The VAS is divided into four levels, with higher scores indicating more severe pain: 0 indicates no pain, 1–3 indicates mild pain, 4–6 indicates moderate pain, and 7–10 indicates severe pain. The follow-up period for VAS assessment was 14 months postoperatively.

Quality of Life: The Oswestry Disability Index (ODI) was used to assess the patients’ quality of life and functional impairment. The ODI includes 10 functional indicators related to daily activities, with each item scoring a maximum of 5 points. The total score is calculated as: ODI = (total score/50) × 100%. A higher percentage indicates greater functional disability. The follow-up period for the ODI assessment was also 14 months postoperatively.

Radiological Parameters: Radiological parameters included the lumbar sagittal Cobb angle, lumbar coronal Cobb angle, and the apical vertebra deviation. These measurements were recorded at the 14-month postoperative follow-up.

Fusion and Collapse Rates: Fusion and collapse rates were evaluated at 6 months postoperatively. Fusion was categorized as successful or failed, while collapse was defined as a reduction in the height or misalignment of the interbody cage compared to its initial placement.

Time to Ambulation and Length of Hospital Stay: The time to first ambulation after surgery and the total duration of hospital stay were recorded for both groups. This included the time patients took to get out of bed for the first time and their overall hospitalization period during treatment.

Complication Rates: Postoperative complications were documented for both groups at the 1-month follow-up. The complication rate was analyzed to compare the safety profiles of the two surgical techniques.

### Statistical analysis

Statistical analysis was performed using SPSS version 27.0. Continuous variables that followed a normal distribution were presented as mean ± standard deviation (± SD), and comparisons between groups were conducted using an independent sample t-test. For continuous variables that did not conform to a normal distribution, data were expressed as median and interquartile range [M (P25, P75)], and the Wilcoxon rank-sum test was employed for group comparisons. Categorical variables were described using frequencies and percentages [n (%)], with comparisons between groups carried out using the chi-square (χ²) test. For repeated measures data, repeated measures analysis of variance (ANOVA) was applied. All statistical tests were two-tailed, and a p-value of less than 0.05 was considered indicative of statistical significance.

## Results

### Demographic and clinical characteristics of patients

The study included a total of 133 patients, with 68 in the OLIF group and 65 in the TLIF group. The demographic and clinical characteristics between the two groups were comparable. The mean age of the patients in the OLIF group was 59.8 ± 7.9 years, while in the TLIF group, it was slightly higher at 61.5 ± 9.8 years. There was a balanced gender distribution across both groups, with 39 males and 29 females in the OLIF group, and 37 males and 28 females in the TLIF group. Regarding neural symptoms, there was a notable difference in the distribution of unilateral and bilateral symptoms between the groups. In the OLIF group, 31 patients experienced unilateral neural symptoms, while in the TLIF group, only 9 patients presented with this condition. Conversely, bilateral neural symptoms were more prevalent in the TLIF group, affecting 54 patients, compared to 34 patients in the OLIF group. Both groups displayed similar distributions of surgical segments. In the OLIF group, the most common surgical segment was L4/5 (*n* = 33), followed by L5/S1 (*n* = 31) and L3/4 (*n* = 4). Similarly, in the TLIF group, L4/5 was the most frequently treated level (*n* = 30), followed by L5/S1 (*n* = 32) and L3/4 (*n* = 3) (Table 1). In summary, the demographic and clinical characteristics of the patients in both the OLIF and TLIF groups were generally well-matched, with minor differences in the distribution of neural symptoms. These similarities ensure that any differences in surgical outcomes are likely due to the procedural approach rather than baseline characteristics.

**Table 1:** Demographic and clinical characteristics of patients in the OLIF and TLIF groups.

Characteristic	OLIF group (*n* = 68)	TLIF group (*n* = 65)
Age (years)	59.8 ± 7.9	61.5 ± 9.8
Gender (n)		
Male	39	37
Female	29	28
Neural symptom (n)		
None	3	2
Unilateral	31	9
Bilateral	34	54
Surgical segment (n)		
L3/4	4	3
L4/5	33	30
L5/S1	31	32

OLIF, Oblique Lateral Interbody Fusion; TLIF, Transforaminal Lumbar Interbody Fusion.

### Pain and functional outcomes in OLIF and TLIF groups

A comparison of pain and functional outcomes between the OLIF and TLIF groups was conducted using the VAS for pain and the ODI for functional impairment. Both groups showed significant improvements in postoperative VAS and ODI scores compared to preoperative levels. Preoperative VAS scores were comparable between the groups (7.46 ± 1.45 in the OLIF group vs. 7.65 ± 1.35 in the TLIF group, *P* = 0.44). Postoperatively, both groups demonstrated substantial reductions in pain, with the OLIF group achieving a VAS score of 5.77 ± 1.32 and the TLIF group reaching 5.49 ± 1.22. However, no statistically significant difference was observed between the two groups in postoperative pain scores (*P* = 0.21). At the last follow-up, the OLIF group exhibited slightly better pain relief with a VAS score of 1.99 ± 0.67 compared to 2.29 ± 0.92 in the TLIF group (*P* = 0.03), indicating a statistically significant difference in long-term pain reduction favoring OLIF (Table 2).

**Table 2:** Comparison of VAS and ODI between the OLIF and TLIF groups (mean ± SD).

Group	Preoperative VAS	Postoperative VAS	Last follow-up VAS	Preoperative ODI (%)	Postoperative ODI (%)	Last follow-up ODI (%)
OLIF group (*n* = 68)	7.46 ± 1.45	5.77 ± 1.32	1.99 ± 0.67	46.75 ± 8.71	34.68 ± 7.65	24.15 ± 6.92
TLIF group (*n* = 65)	7.65 ± 1.35	5.49 ± 1.22	2.29 ± 0.92	48.82 ± 7.97	33.74 ± 8.14	25.77 ± 5.85
t-value	0.78	1.27	2.16	1.43	0.69	1.46
P-value	0.44	0.21	0.03	0.16	0.49	0.15

VAS, Visual Analogue Scale; ODI, Oswestry Disability Index; OLIF, Oblique Lateral Interbody Fusion; TLIF, Transforaminal Lumbar Interbody Fusion.

Regarding functional outcomes, preoperative ODI scores were also similar between the groups (46.75 ± 8.71 in the OLIF group vs. 48.82 ± 7.97 in the TLIF group, *P* = 0.16). Postoperatively, both groups experienced improvements in functional status. The OLIF group had a postoperative ODI of 34.68 ± 7.65, while the TLIF group scored 33.74 ± 8.14, with no significant difference between them (*P* = 0.49). At the last follow-up, the OLIF group showed slightly better functional outcomes, with an ODI of 24.15 ± 6.92 compared to 25.77 ± 5.85 in the TLIF group, though this difference did not reach statistical significance (*P* = 0.15) (Table 2). In summary, both OLIF and TLIF provided effective pain relief and improved functional outcomes, with OLIF demonstrating a slight advantage in long-term pain reduction. However, functional outcomes were comparable between the two surgical techniques.

### Radiological outcomes in OLIF and TLIF groups

A comparison of radiological outcomes between the OLIF and TLIF groups demonstrated significant improvements in all measured imaging parameters, including sagittal and coronal Cobb angles, as well as apical vertebra deviation. Both surgical techniques were effective in correcting spinal alignment, although there were notable differences in the degree of correction achieved.

**Fig. 1:**
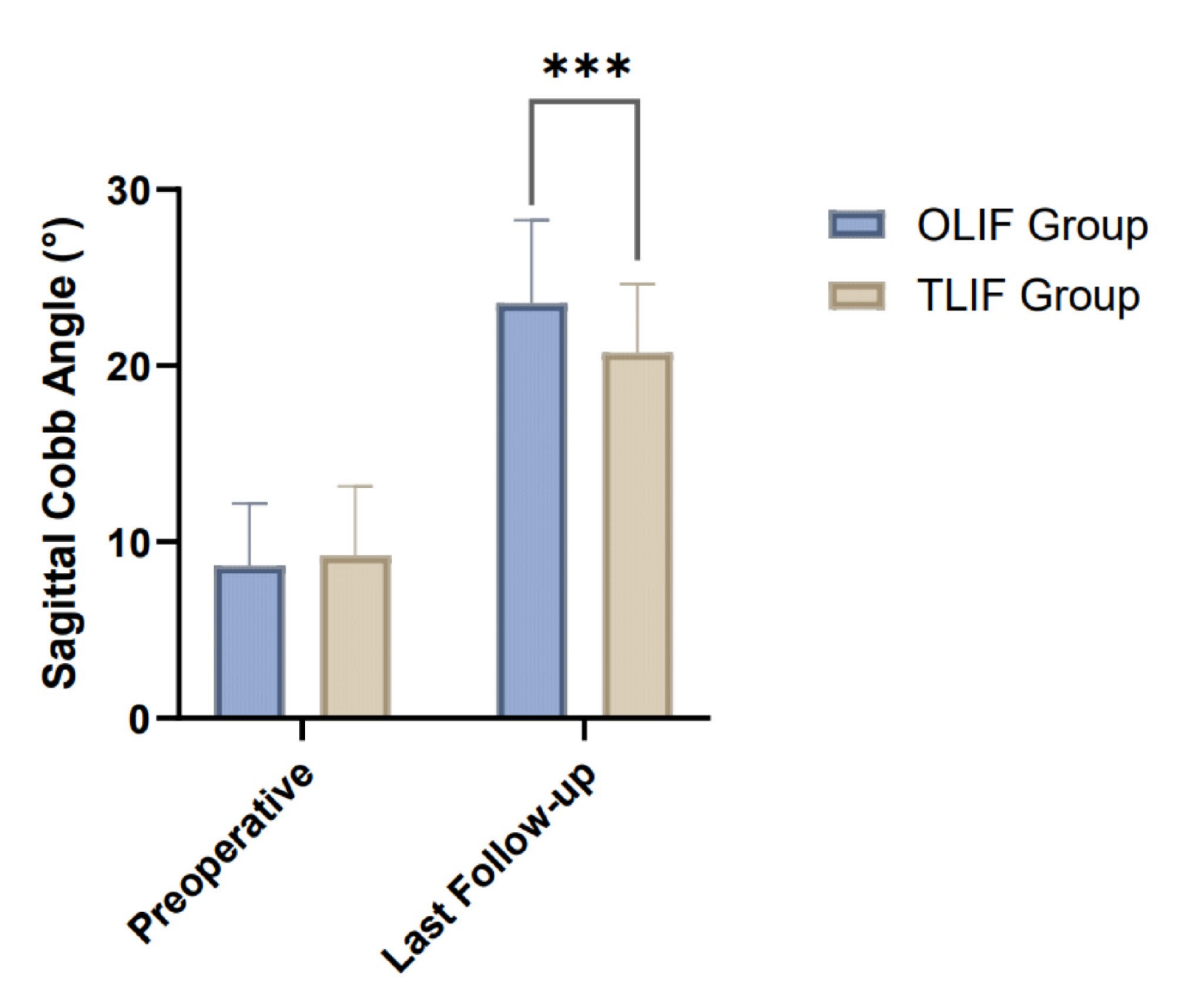
Comparison of Sagittal Cobb Angle between the OLIF and TLIF groups at preoperative and last follow-up.

**Fig. 2:**
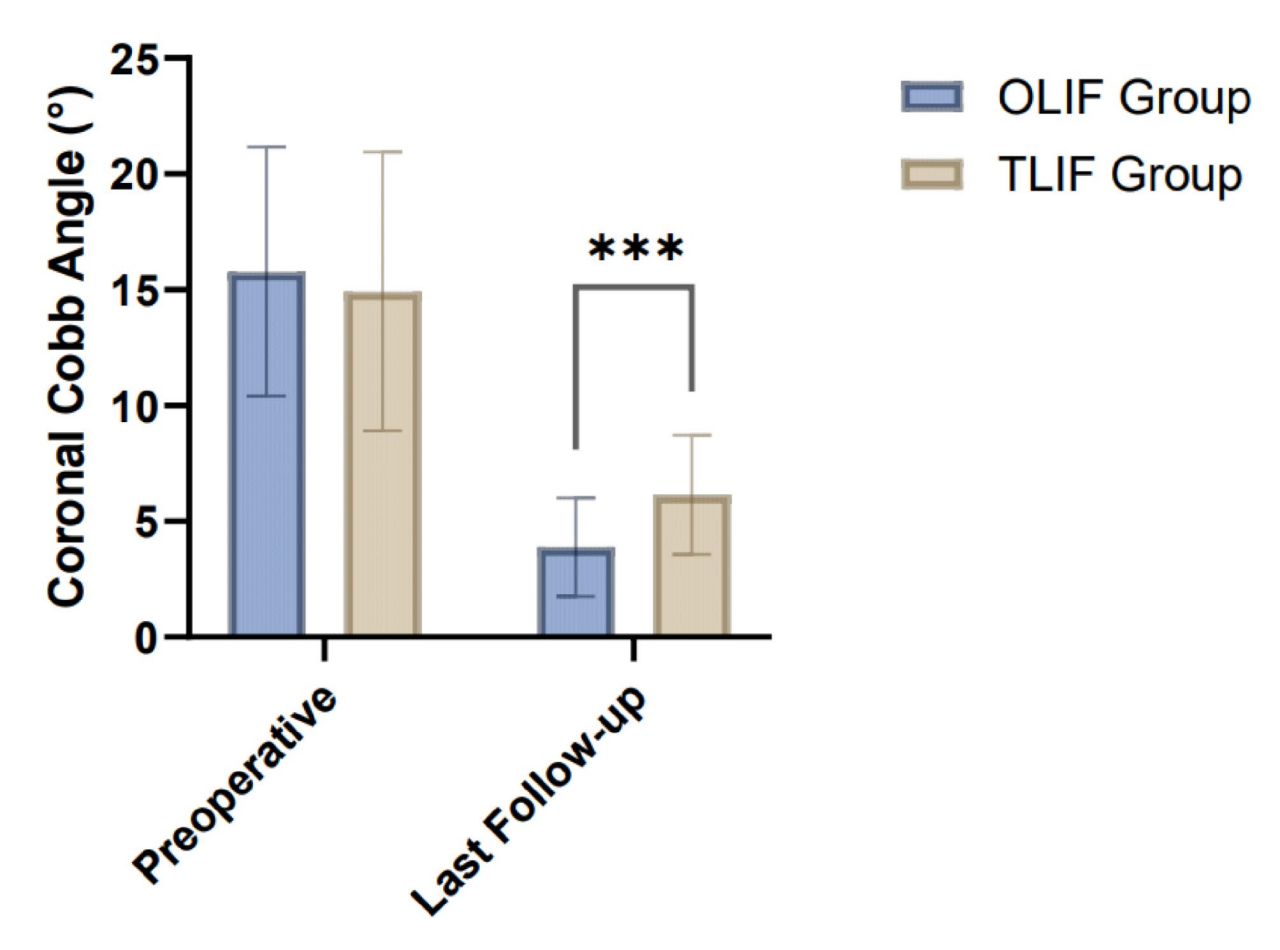
Comparison of Coronal Cobb Angle between the OLIF and TLIF groups at preoperative and last follow-up.

**Fig. 3:**
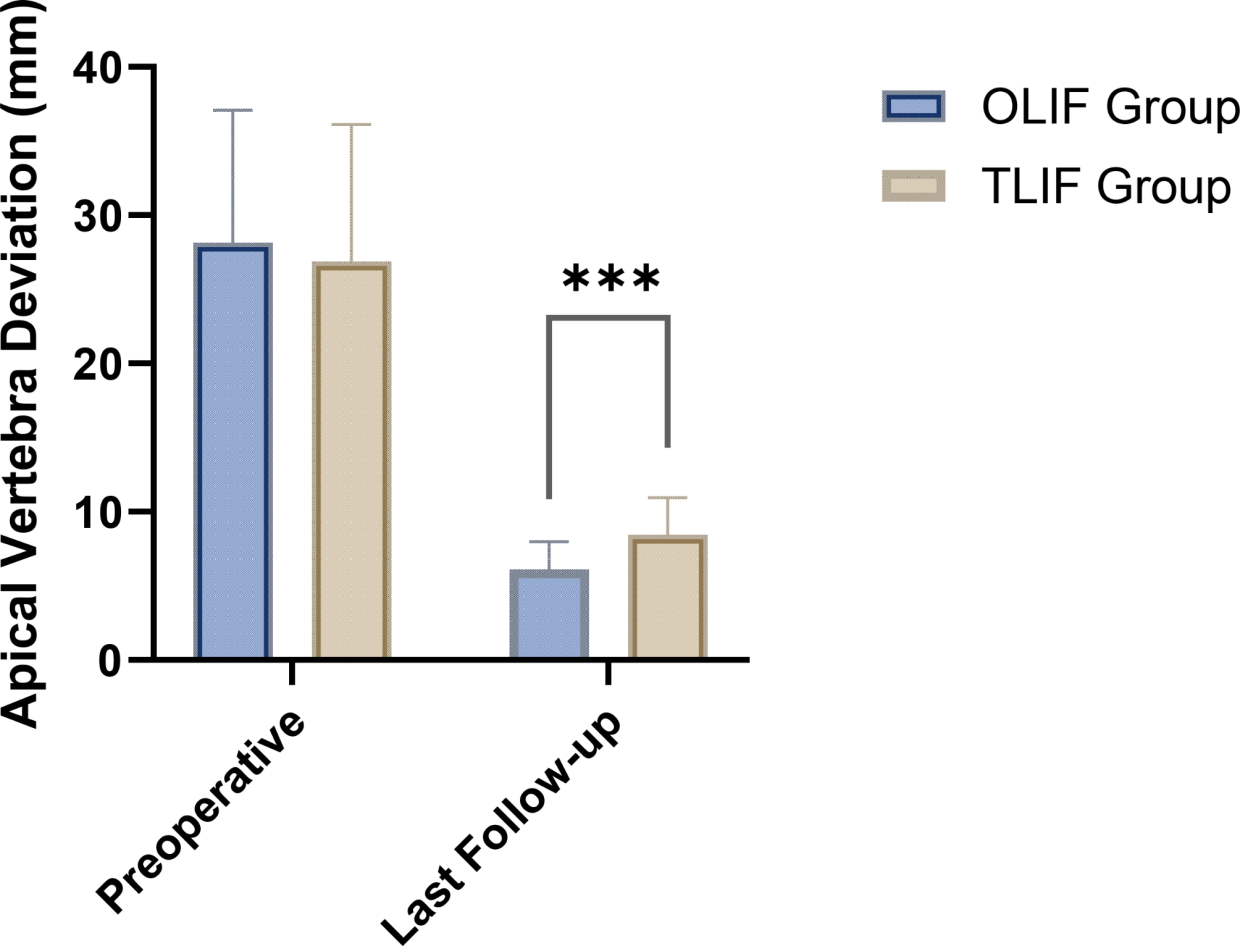
Comparison of Apical Vertebra Deviation between the OLIF and TLIF groups at preoperative and last follow-up.

The preoperative sagittal Cobb angle was comparable between the two groups (8.67 ± 3.52° in the OLIF group vs. 9.23 ± 3.94° in the TLIF group, *P* = 0.39). At the last follow-up, both groups showed significant improvements in the sagittal Cobb angle. The OLIF group exhibited a larger correction to 23.55 ± 4.72°, while the TLIF group improved to 20.75 ± 3.88°. The difference in the last follow-up values between the two groups was statistically significant (*P* < 0.001), indicating greater sagittal alignment correction in the OLIF group (Table 3; Fig. 1). Similarly, for coronal Cobb angle correction, both groups achieved significant reductions from preoperative levels (15.78 ± 5.38° to 3.89 ± 2.13° in the OLIF group and 14.92 ± 6.02° to 6.15 ± 2.57° in the TLIF group). The OLIF group demonstrated superior correction, with a significant difference at the last follow-up compared to the TLIF group (*P* < 0.001) (Table 3; Fig. 2). Apical vertebra deviation also showed marked improvement in both groups. Preoperatively, the OLIF group had an average deviation of 28.15 ± 8.92 mm, which decreased to 6.12 ± 1.85 mm at the last follow-up. Similarly, the TLIF group showed a reduction from 26.89 ± 9.21 mm to 8.45 ± 2.49 mm. The OLIF group achieved a greater degree of correction, as reflected by the significant difference between the groups at the last follow-up (*P* < 0.001) (Table 3; Fig. 3).

**Table 3:** Comparison of imaging parameters between the OLIF and TLIF groups (mean ± SD).

Group	Preoperative	Last follow-up	t-value	*P*-value
Sagittal Cobb angle (°)				
OLIF group (*n* = 68)	8.67 ± 3.52	23.55 ± 4.72	20.67	< 0.001
TLIF group (*n* = 65)	9.23 ± 3.94	20.75 ± 3.88	16.80	< 0.001
t-value	0.87	3.73		
P-value	0.39	< 0.001		
Coronal Cobb angle (°)				
OLIF group (*n* = 68)	15.78 ± 5.38	3.89 ± 2.13	16.9	< 0.001
TLIF group (*n* = 65)	14.92 ± 6.02	6.15 ± 2.57	10.8	< 0.001
t-value	0.87	5.53		
P-value	0.39	< 0.001		
Apical vertebra deviation (mm)				
OLIF group (*n* = 68)	28.15 ± 8.92	6.12 ± 1.85	19.94	< 0.001
TLIF group (*n* = 65)	26.89 ± 9.21	8.45 ± 2.49	15.58	< 0.001
t-value	0.80	6.14		
P-value	0.42	< 0.001		

OLIF, Oblique Lateral Interbody Fusion; TLIF, Transforaminal Lumbar Interbody Fusion.

Both OLIF and TLIF were effective in achieving spinal realignment, with the OLIF group showing significantly better results in correcting sagittal and coronal Cobb angles as well as apical vertebra deviation at the last follow-up. These findings suggest that OLIF may provide superior radiological outcomes in the treatment of lumbar disc herniation.

### Fusion and collapse rates in OLIF and TLIF groups

In evaluating the fusion and collapse rates between the OLIF and TLIF groups, both surgical techniques demonstrated high fusion success, but significant differences were observed in the collapse rates. The fusion rate in the OLIF group was slightly higher at 97.92% (94 out of 96 patients), compared to 96.61% (114 out of 118 patients) in the TLIF group. Despite this small difference, statistical analysis revealed no significant difference between the two groups in terms of fusion success (*P* = 0.56). Both techniques appear highly effective in achieving solid fusion in patients with lumbar disc herniation. However, when comparing collapse rates, a significant difference was observed. The OLIF group had a much lower collapse rate of 8.33% (8 out of 96 patients) compared to 18.64% (22 out of 118 patients) in the TLIF group. The difference in collapse rates was statistically significant (*P* < 0.001), indicating a higher risk of cage subsidence or collapse in the TLIF group compared to the OLIF group (Table 4). In conclusion, while both OLIF and TLIF achieved similarly high fusion rates, OLIF showed a significantly lower collapse rate, suggesting that it may offer an advantage in terms of implant stability and long-term structural integrity in patients undergoing lumbar interbody fusion.

**Table 4:** Comparison of Fusion Rate and Collapse Rate between the OLIF and TLIF groups (n, %).

Group	Fusion rate (%)	Collapse rate (%)
OLIF group (*n* = 96)	94 (97.92)	8 (8.33)
TLIF group (*n* = 118)	114 (96.61)	22 (18.64)
χ^2^-value	0.33	10.76
P-value	0.56	< 0.001

OLIF, Oblique Lateral Interbody Fusion; TLIF, Transforaminal Lumbar Interbody Fusion.

### Postoperative recovery

**Fig. 4:**
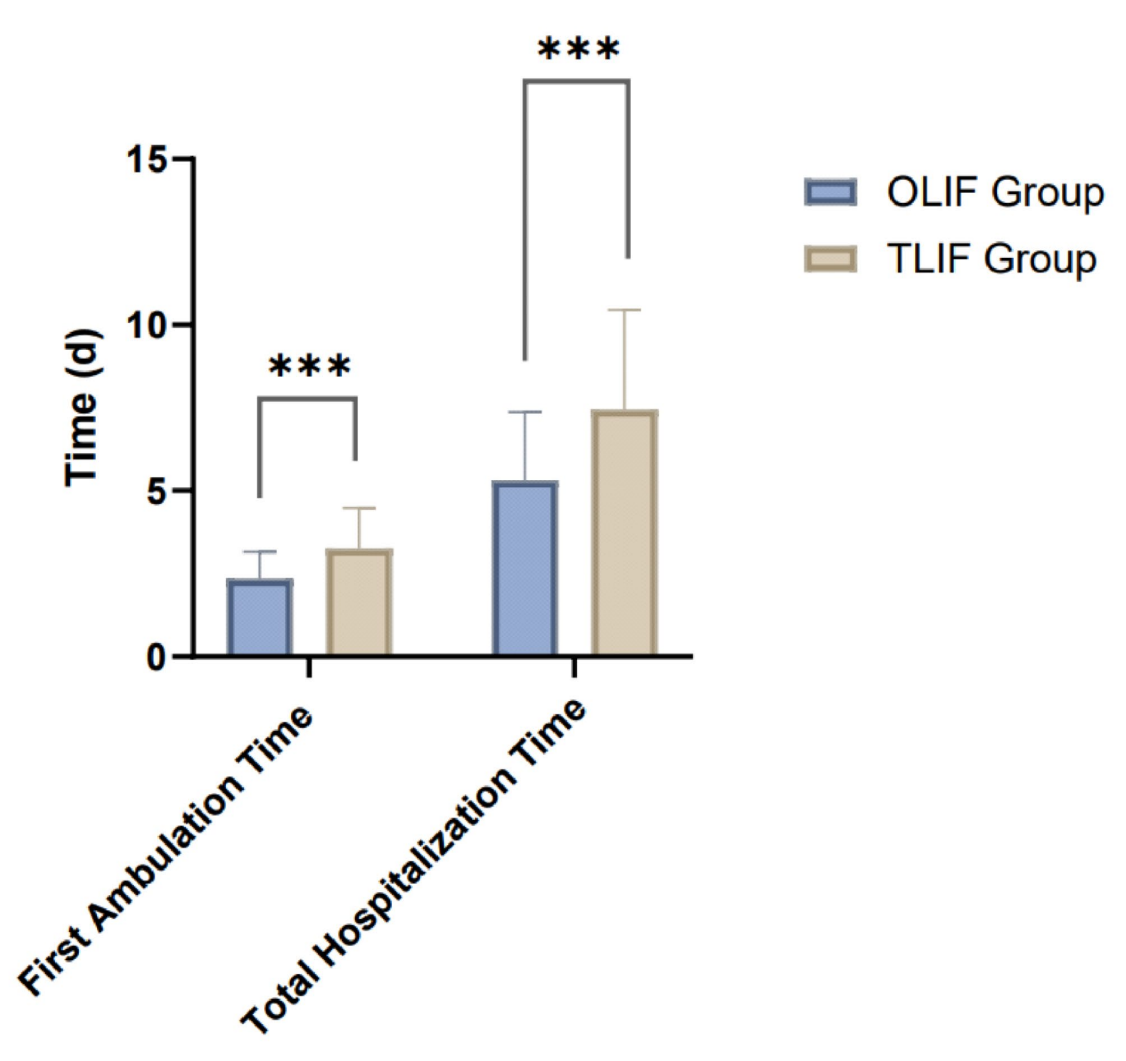
Comparison of fusion and collapse rates between the OLIF and TLIF groups.

The comparison of postoperative recovery between the OLIF and TLIF groups, measured by first ambulation time and total hospitalization duration, revealed significant differences favoring the OLIF technique. Patients in the OLIF group demonstrated faster recovery, with a mean time to first ambulation of 2.35 ± 0.81 days compared to 3.27 ± 1.21 days in the TLIF group. This difference was statistically significant (*P* < 0.001), indicating that patients undergoing OLIF were able to mobilize earlier after surgery. Similarly, total hospitalization time was shorter for the OLIF group, with an average stay of 5.32 ± 2.05 days, compared to 7.45 ± 3.01 days in the TLIF group. The difference in hospitalization duration was also statistically significant (*P* < 0.001), suggesting that patients treated with OLIF required a shorter postoperative hospital stay (Table 5; Fig. 4). In summary, the OLIF group exhibited faster postoperative recovery in terms of earlier ambulation and reduced hospitalization time compared to the TLIF group, indicating potential benefits of the OLIF approach in facilitating quicker rehabilitation and reducing healthcare resource utilization.

**Table 5:** Comparison of first ambulation time and total hospitalization time after operation (d, mean ± SD).

Group	First ambulation time (d)	Total hospitalization time (d)
OLIF group (*n* = 68)	2.35 ± 0.81	5.32 ± 2.05
TLIF group (*n* = 65)	3.27 ± 1.21	7.45 ± 3.01
t-value	5.17	4.79
P-value	< 0.001	< 0.001

OLIF, Oblique Lateral Interbody Fusion; TLIF, Transforaminal Lumbar Interbody Fusion.

### Subgroup analysis by surgical level

Our surgical level-based subgroup analysis (L3/4, L4/5, L5/S1) revealed that while OLIF and TLIF outcomes at the L3/4 level were comparable across VAS pain scores, ODI functional scores, sagittal Cobb angle correction, and hospital stay, OLIF provided significant advantages at the L4/5 and L5/S1 levels, with better long-term pain relief, greater sagittal Cobb angle correction, and shorter hospitalization durations. Although limited by the retrospective design to analyzing outcomes by surgical level, these findings suggest that OLIF may be particularly beneficial for patients undergoing procedures at L4/5 and L5/S1, offering clinically relevant insights for optimal surgical approach selection.

## Discussion

This study presents a comparative analysis of the efficacy of OLIF versus TLIF in managing LDH with accompanying lumbar instability. Surgical intervention becomes essential for LDH cases that are refractory to conservative management. OLIF and TLIF, two widely adopted techniques with distinct surgical approaches, were evaluated for their impact on pain relief, functional improvement, spinal alignment correction, fusion rates, and postoperative recovery. By retrospectively analyzing 133 cases from 2020 to 2022, this study aims to provide evidence-based guidance to inform surgical decision-making in LDH patients. Data was collected on pain scores, functional outcomes, radiographic parameters, fusion success, and recovery metrics. This study adds valuable insights to the existing literature by systematically comparing the outcomes of OLIF and TLIF, which may assist clinicians in selecting the optimal approach for LDH treatment^14,15^. The results of this study provide a comprehensive comparison of the efficacy of OLIF versus TLIF in the treatment of LDH. Both techniques were found to be effective in relieving pain, improving functional outcomes, achieving spinal realignment, and promoting solid fusion. However, notable differences were observed between the two approaches in terms of long-term pain relief, radiological outcomes, implant stability, and recovery times, with OLIF demonstrating certain advantages over TLIF.

Both OLIF and TLIF demonstrated substantial improvements in pain relief and fusion stability for LDH patients, as evidenced by high fusion rates in both groups^16,17^. However, the OLIF group showed a statistically significant advantage in long-term pain reduction (*P* = 0.03), which may stem from the minimally invasive nature of OLIF, minimizing trauma to the posterior spinal structures and nerve roots^18,19^. Furthermore, OLIF had a significantly lower collapse rate (8.33% vs. 18.64% for TLIF, *P* < 0.001), likely attributable to the larger, more stable interbody cages used in this approach^20^. This enhanced stability and reduced risk of implant collapse underscore OLIF’s potential for sustained structural integrity, making it a more favorable option for long-term spinal fusion outcomes, especially in patients with compromised bone quality^21,22^.

Radiological outcomes indicate that OLIF offers superior correction of sagittal and coronal Cobb angles, as well as apical vertebra deviation (*P* < 0.001 for all parameters), due to its lateral trajectory, which allows for larger cages and more precise alignment^23,24^. This control over three-dimensional spinal deformities is advantageous for patients with significant misalignment, a challenge for TLIF’s posterior approach^25,26^. Additionally, OLIF’s muscle-sparing technique contributed to faster postoperative recovery, with patients ambulating sooner and experiencing shorter hospitalization times (*P* < 0.001 for both metrics). This accelerated recovery not only reduces postoperative pain and risk of complications but also optimizes healthcare resource utilization, offering potential cost-effectiveness benefits in systems where hospital stay duration is a consideration^27,28^.

Wang et al.‘s^29^ meta-analysis identified OLIF’s advantages in reducing blood loss, shortening hospital stays, increasing fusion rates, and restoring disc and foraminal heights, with MIS-TLIF showing a lower complication rate. In alignment with our findings, OLIF demonstrated superior pain relief, spinal alignment, and recovery outcomes. Our study builds on these conclusions by providing a focused analysis of collapse rates and long-term pain relief, areas less emphasized by Wang et al. Similarly, Li et al.^30^ reported that OLIF led to less blood loss, shorter hospital stays, and greater improvements in VAS leg pain and ODI scores compared to TLIF, with equivalent fusion rates. Our results corroborate these findings and further underscore OLIF’s advantage in reducing collapse rates and enhancing sagittal alignment, which reinforces its clinical utility in LDH treatment. Shi et al.^31^ also found OLIF superior in surgical time, intraoperative bleeding, hospital duration, and postoperative improvements in VAS and ODI scores, with comparable fusion success. Our study supports these benefits and expands on them by incorporating analyses of collapse rates and ambulation time, offering a more comprehensive view of OLIF’s impact on stability and recovery. These comparisons integrate our findings with current literature, underscoring our study’s contribution to the understanding of OLIF’s clinical advantages in LDH.

A post hoc power analysis was conducted to validate the adequacy of our sample size. Using sagittal Cobb angle correction as a representative outcome variable, we calculated a pooled standard deviation of 4.32 and an effect size of 0.648, requiring a sample of 77 patients (39 per group) to achieve 80% power with a significance level of 0.05. Our actual sample size of 133, therefore, exceeds this minimum requirement, confirming that our study is sufficiently powered to detect significant differences in primary outcomes. This substantiates the robustness of our findings within a retrospective design. Retrospective studies are inherently prone to baseline imbalances due to historical selection bias, reflected here in the uneven distribution of unilateral versus bilateral symptoms. However, our primary outcomes—postoperative pain reduction, functional improvement, spinal alignment correction, and fusion rates—are unaffected by neurological laterality, minimizing the impact of this imbalance on our conclusions. Regarding fusion assessment, based on clinical experience and literature, evaluating fusion success at 6 months is a valid initial benchmark^32^. Our data show that by this point, the majority of patients had achieved successful fusion, providing a reliable early measure of surgical efficacy. These factors collectively support the validity of our results, while future studies could enhance findings with prospective design and extended follow-up.

This study highlights OLIF’s potential benefits over TLIF in managing lumbar disc herniation (LDH), especially in terms of long-term pain relief, spinal alignment correction, lower collapse rates, and expedited recovery. Nonetheless, our study has important limitations. The retrospective design introduces selection bias and baseline variability, impacting generalizability. Additionally, although our sample size was sufficient for primary outcomes, it is relatively modest, and the follow-up period limits insights into long-term complications, such as adjacent segment degeneration (ASD) and alignment maintenance. Technical limitations also shape patient selection and outcomes in OLIF and TLIF. OLIF’s lateral approach minimizes muscle dissection and blood loss, supporting quicker recovery but is anatomically restricted at lower lumbar levels, particularly L5/S1, often requiring posterior fixation for stability. Conversely, TLIF’s posterior access allows thorough decompression and fusion but necessitates more paraspinal dissection, which can lead to higher postoperative pain and extended recovery, as well as a higher risk of subsidence, as indicated by our collapse rate findings. Future studies should include prospective designs, larger sample sizes, and extended follow-up to capture long-term durability, including ASD and fusion maintenance, while also incorporating cost-effectiveness and safety analyses. This will support refined patient selection criteria, enabling tailored surgical approaches to optimize LDH management outcomes.

## Conclusions

In conclusion, both OLIF and TLIF could be effective surgical techniques for treating lumbar disc herniation, offering substantial pain relief and functional improvement. However, OLIF might demonstrate advantages in achieving better long-term pain reduction, enhanced spinal alignment correction, and faster recovery. These findings suggest that OLIF could be a more favorable option for patients requiring lumbar interbody fusion, though further prospective studies are warranted to confirm these potential benefits.

## Data Availability

The datasets used and/or analyzed during the present study are available from the corresponding author on reasonable request.
